# Questions & Controversies

**DOI:** 10.1038/cddiscovery.2015.39

**Published:** 2015-10-19

**Authors:** J Silke

**Affiliations:** 1 The Walter and Eliza Hall Institute of Medical Research, Parkville, Victoria 3052, Australia; 2 Department of Medical Biology, University of Melbourne, Parkville, Victoria 3050, Australia

*Cell Death Discovery* is introducing a new series of articles titled Questions & Controversies (Q&C). The authors of these articles have been asked to identify important questions in their research field. In some cases the question arises because of two different sets of data or different interpretations of similar data. For such controversies the authors should try and identify the substantive reasons for the disagreement and where they can, suggest experiments that might help to definitively support or refute one interpretation or the other.

The motivation behind introducing this format for a review article is several fold. First, we believe that this is a quintessentially scientific approach. Science, when it is true to its own rules, challenges preconceived ideas and, if it cannot find support for them, disposes of them. However, because scientists are investigating the unknown there will always be missteps along the way. This is something that the earliest proponents of a ‘scientific method’, such as Francis Bacon, recognised very well: ‘Truth will sooner come out from error than from confusion’. The goal however is to let the data speak as Thomas Huxley, Darwin's Bulldog, famously said: ‘Science is organized common sense where many a beautiful theory was killed by an ugly fact.’

Second, while other reviews seek to give an overview of the research area and occasionally mention areas of controversy, it is our belief that research in these areas will move forward more quickly if controversies are highlighted. Controversies help distil problems down to their fundamental essence and thereby already point to ways to find a solution. Stating a problem clearly is the first step on the road to its resolution and it is something that scientists have been doing forever. Controversies also usually swirl around points of particular importance to a field; it is hard to imagine a controversy about whether dawn or dusk is the most beautiful time of day.

Third, we believe it will be helpful to have a forum for these discussions. This series therefore seeks to acknowledge and embrace the facts that there are and should be disagreements in research, but also to provide a scientific framework and forum so that these disagreements can form the basis for the next advance in our understanding. These are not new ideas: the history of scientific research is a history of disagreements that led to greater understanding. Scientific disagreements are not resolved by money or the faith of the proponents, but by data. This series aims to try and encourage such an objective scientific dialectic.

Therefore, if you find your research highlighted in one of these Q&C pieces, pat yourself on the back. You are clearly working in an exciting area at the forefront of knowledge where there is a passion for discovery. But bear in mind that you may be wrong and ask yourself how you can show that you are wrong: that is what a scientist would try and do. Hopefully this series might give you some ideas to test.

